# The association of salivary biomarkers with self-compassion and perfectionism in medical students exposed to pre-exam stress

**DOI:** 10.1192/j.eurpsy.2025.755

**Published:** 2025-08-26

**Authors:** P. Boonyalug, R. Nabhindhakara, P. Paradeevisut, P. Tayawitid, R. Settacomkul, T. Prachason, P. Vivithanaporn

**Affiliations:** 1 Faculty of Medicine Ramathibodi Hospital, Bangkok; 2 Chakri Naruebodindra Medical Institute (CNMI), Faculty of Medicine Ramathibodi Hospital, Samut Prakan; 3 Psychiatry, Faculty of Medicine Ramathibodi Hospital, Bangkok, Thailand

## Abstract

**Introduction:**

Evidence has shown that perfectionism is linked with increased perceived stress, whereas self-compassion might mitigate poor outcomes related to stress. However, how these traits influence stress responses in a naturalistic setting is unclear.

**Objectives:**

The study aims to test the associations of perfectionism and self-compassion traits with stress-related biomarkers, namely C-reactive protein (CRP), alpha-amylase, and cortisol, in medical students exposed to pre-exam stress.

**Methods:**

61 second-year medical students were enrolled in this study. At baseline, perfectionism and self-compassion were self-rated using the Self-Compassion Scale and the Short-Revised Almost Perfect Scale, respectively. Morning saliva samples were collected at baseline and one week before the exam. The levels of salivary CRP, alpha-amylase, and cortisol were quantified as biomarkers for inflammation, sympathetic activity, and hypothalamus-pituitary-adrenal axis, respectively, using enzyme-linked immunosorbent assay. Multiple linear regression analysis was performed to test the associations between the two traits with pre-exam salivary biomarkers, adjusted for baseline salivary biomarkers, age, sex, and body mass index (BMI). Other potential confounding variables, including acute illness, underlying mood disorder, and lifestyle factors, were also added to the model as sensitivity analyses.

**Results:**

Adjusted for the baseline level of biomarker, age, sex, and BMI, perfectionism traits significantly predicted pre-exam salivary alpha-amylase (B = 0.04, 95% CI 0.01 to 0.07, p = .007), but not CRP (B = -0.03, 95% CI -0.07 to 0.01, p = .177) or cortisol (B = 0.004, 95% CI -0.005 to 0.012, p = .424). No significant associations were found between self-compassion traits and the pre-exam levels of all three salivary biomarkers. The sensitivity analysis, additionally adjusted for other potential confounding factors, confirmed the significant positive association between perfectionism traits and pre-exam salivary alpha-amylase (B = 0.04, 95% CI 0.01 to 0.07, p = .006).

**Conclusions:**

Perfectionism traits could positively predict the level of morning salivary alpha-amylase in naturalistic stress exposure, suggesting a heightened sympathetic activity among those with high perfectionism in response to stress. Replication studies in a larger sample with more diverse populations are warranted.

**Disclosure of Interest:**

None Declared

